# Non-invasive prediction of prognostic immune subtypes in lung adenocarcinoma using PET/CT-based radiomics

**DOI:** 10.3389/fonc.2026.1739125

**Published:** 2026-04-29

**Authors:** Sijia Zhang, Yang Wang, Dong Dai, Wengui Xu

**Affiliations:** 1 Department of Molecular Imaging and Nuclear Medicine, Tianjin Medical University Cancer Institute and Hospital, National Clinical Research Center of Cancer, Key Laboratory of Cancer Prevention and Therapy, Tianjin, China; 2 Department of Biotherapy, Tianjin Medical University Cancer Institute and Hospital, National Clinical Research Center of Cancer, Key Laboratory of Cancer Prevention and Therapy, Tianjin, China

**Keywords:** 18F-FDG PET/CT, immune related subtypes, lung adenocarcinoma, radiomics, transcriptomic

## Abstract

**Background:**

The treatment and prognosis of lung adenocarcinoma (LUAD) remain major clinical challenges. While transcriptomic profiling can define immune subtypes, its invasive nature limits clinical utility.

**Objectives:**

We aimed to bridge this translational gap by developing and validating a non-invasive ^18^F-FDG PET/CT radiomics-based biomarker to predict these subtypes and assess their therapeutic relevance.

**Methods:**

We performed consensus clustering on transcriptomic data from 773 LUAD patients (TCGA/GEO) to identify novel immune subtypes, developed a radiomics-based machine learning classifier using their PET/CT data (n=115) and conducted a biologically and clinically supported external evaluation in an independent cohort from Tianjin Medical University Cancer Hospital (TMUCIH, n=249). In this cohort, we compared immune patterns with transcriptomic subtypes via immunohistochemical staining, and their prognostic significance was evaluated in patients receiving EGFR tyrosine kinase inhibitors (n=118) and immune checkpoint inhibitors (n=75).

**Results:**

We identified three robust immune subtypes (log-rank p=0.0017): Inflammatory (ClusterA), Activated (ClusterB), and Evasive (ClusterC). Our radiomics model predicted these subtypes with high accuracy (one-vs-rest AUC = 0.84). Immunohistochemical patterns were consistent with predicted subtypes and stratified overall survival in both the TKI-treated (log-rank p-value=0.041) and ICI-treated (log-rank p-value =0.024) cohorts. These results support a validated, non-invasive approach linking genomics and radiomics to enhance patient stratification and guide personalized treatment strategies in LUAD.

## 1 Introduction

Lung cancer is the leading cause of cancer-related death worldwide, with non-small cell lung cancer (NSCLC), particularly lung adenocarcinoma (LUAD), being the most prevalent subtype (1). While the advent of targeted therapies against driver mutations (e.g., EGFR) and immune checkpoint inhibitors (ICIs) has revolutionized treatment, the prognosis for advanced LUAD remains challenging due to primary or acquired resistance. This clinical heterogeneity highlights the urgent need for refined predictive biomarkers and effective patient stratification strategies (2).

The tumour microenvironment (TME) is a critical determinant of tumour progression and therapeutic response (3). Efforts to characterize the TME have led to valuable frameworks, such as the “immune hot, altered, and cold” paradigm, and more nuanced immune archetypes (4, 5). Although high-throughput genomic profiling provides a powerful way to molecular classification, its reliance on invasive tissue biopsies introduces sampling bias and limits repeat analysis. This demands a non-invasive methods capable of accurately recapitulating biologically-defined subtypes (6).

Radiomics, the high-throughput extraction of quantitative features from medical images, offers a powerful solution to this challenge. Fluorine-18 Fluorodeoxyglucose Positron Emission Tomography/Computed Tomography (^18^F-FDG PET/CT) imaging is particularly well-suited for this approach, as the metabolic signature it captures (the Warburg effect) is intrinsically linked to the acidic, immunosuppressive TME (7–9) Therefore, the signature captured by ^18^F-FDG PET/CT imaging provides a strong biological rationale for immunophenotyping (10–12).

**Figure 1:**
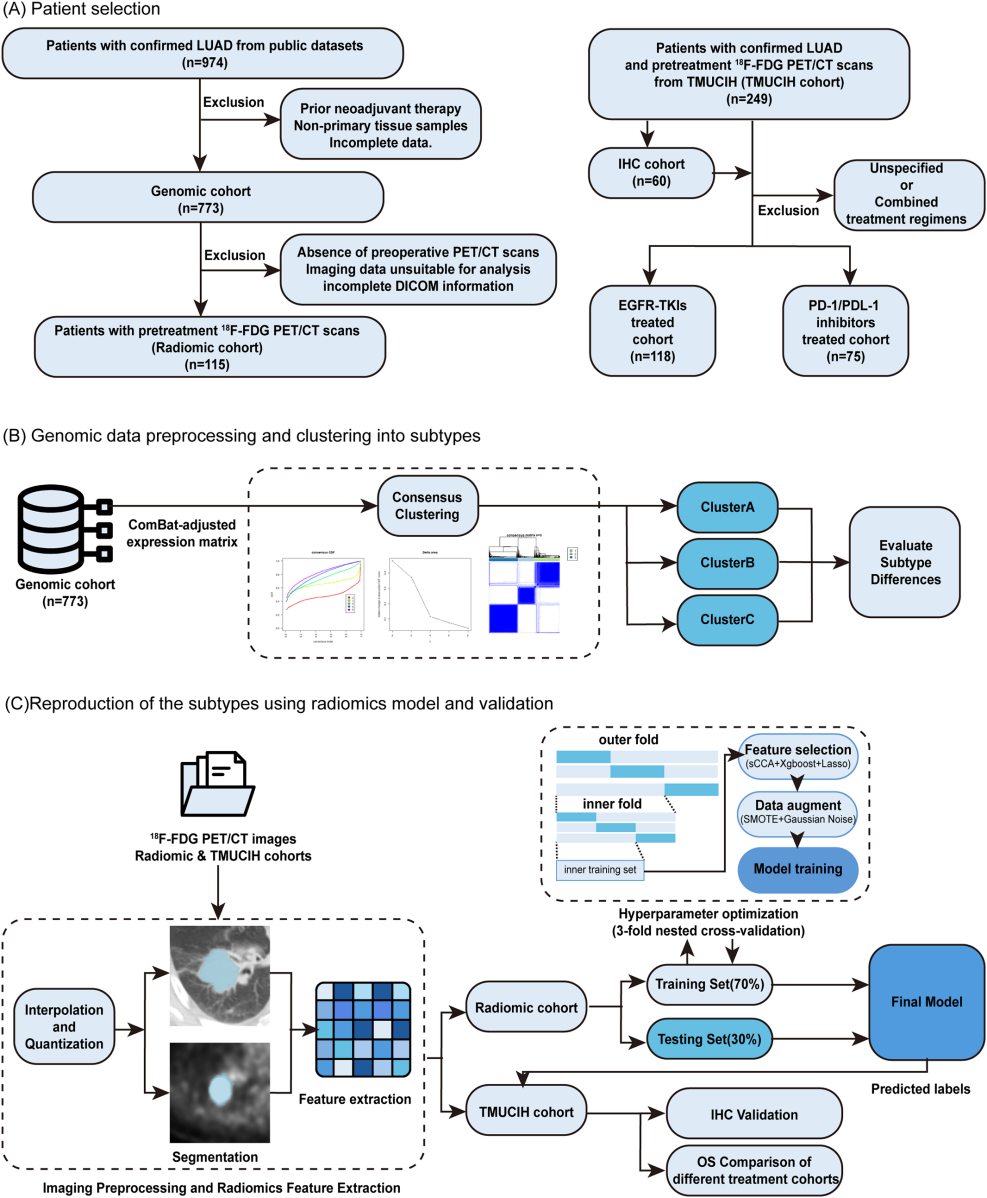
Overview of the study pipeline. **(A)** Establishment of public cohort and TMUCIH cohort. **(B)** Identification of transcriptomic subtypes based on genetic signatures. **(C)** Radiomics workflow, including radiomic features extraction, deep-learning model construction and clinical efficacy evaluation.

In this study, we hypothesized that novel LUAD immune subtypes, defined by unbiased transcriptomic clustering and distinct TME characteristics, could be non-invasively predicted using radiomic features extracted from pretreatment ^18^F-FDG PET/CT scans. Accordingly, this study was designed to identify novel immune subtypes in LUAD using transcriptomic clustering, develop and validate a radiomic model to non-invasively predict these subtypes, and finally, assess the prognostic and predictive significance of this classification system across distinct therapeutic cohorts (**Figure 1**).

## 2 Materials and methods

### 2.1 Data acquisition and patient cohorts

This study adhered to the Declaration of Helsinki. For the publicly available data, the study was deemed exempt from Institutional Review Board (IRB) review by the Ethics Committee of Tianjin Medical University Cancer Hospital. For the local data, the requirement for informed consent was waived by the same Ethics Committee because this was a retrospective analysis and used anonymized data. The following two cohorts were established and used for the analysis (**Supplementary Table 1**):

1. A cohort of lung adenocarcinoma patients was assembled from publicly available datasets, including the Cancer Genome Atlas (TCGA), Clinical Proteomic Tumour Analysis Consortium 3 (CPTAC-3), and GSE103584 from the Gene Expression Omnibus (GEO). RNA-seq data and clinical data were acquired from the Genomic Data Commons and Gene Expression Omnibus. Patients were excluded if they had received prior neoadjuvant therapy, provided non-primary tissue samples, or had incomplete expression data. Corresponding ^18^F-FDG PET/CT data were downloaded from The Cancer Imaging Archive (TCIA). Patients were excluded if they lacked preoperative or pretreatment PET/CT scans, had imaging quality unsuitable for analysis, or presented with incomplete DICOM information. In total, our study ultimately included 773 patients and 115 PET/CT scans (13–19).
2. A cohort of lung adenocarcinoma patients from Tianjin Medical University Cancer Hospital (TMUCIH) underwent diagnostic ^18^F-FDG PET/CT scans within one month before diagnosis from 2009 to 2023. Exclusion criteria included patients receiving any anti-tumour treatment or invasive examination before the PET/CT scan and image quality unsuitable for analysis. A total of 249 patients were enrolled, in which 60 patients with available tissue were randomly selected as IHC cohort for immunohistochemical staining to further validate the subtype immune signatures. They were further divided in two subcohorts, the first was the EGFR tyrosine kinase inhibitors treated cohort (TKI-treated cohort), comprising 118 patients diagnosed with EGFR-mutant lung adenocarcinoma between 2011 and 2020. All patients subsequently received TKI therapy, regardless of whether they had undergone lobectomy. The median follow-up was 42.9 months. The second was the PD-1/PD-L1 inhibitors treated cohort (ICI-treated cohort), consisting of 75 patients with EGFR wildtype lung adenocarcinoma diagnosed via biopsy between 2018 and 2023. They received ICI monotherapy or combination chemotherapy. The median follow-up time was 28.3 months. Overall survival (OS) was followed up from the date of definitive pathological diagnosis until death or loss to follow-up due to various causes. Patients with missing or inaccessible information in the electronic medical record system, or those who received unspecified or combined treatment regimens, were excluded from these subcohorts.

### 2.2 Transcriptomic analysis and subtype identification

We acquired transcriptomic data (TPM values) from the three public cohorts using the R packages “TCGAbiolinks” and “GEOquery”. All RNA-seq data were merged by Ensembl Gene ID. Genes with a TPM expression greater than 1 in more than 50% of the samples were retained, and the filtered data were then log2-transformed [log2(TPM + 1)]. Batch effects were eliminated using the Combat algorithm. Unsupervised cluster analysis was performed using the “ConsensusClusterPlus” package, employing the K-means algorithm (20). The optimal and robust number of clusters were assessed using cumulative distribution functions (CDFs) curve, Delta Area curve, consensus matrices, and principal component analysis (PCA). Pairwise comparisons of the identified subtypes were conducted using the “limma” package to identify differential gene expression (DEG) common to at least two subtypes. Benjamini-Hochberg (BH) false discovery rate (FDR) correction was applied, with a significance threshold of adjusted p-value< 0.05 and an absolute log2 fold change (|log2(FC)|) > 1. Gene ontology (GO) and Kyoto Encyclopedia of Genes and Genomes (KEGG) were used for systematic annotation and enrichment analysis. Kaplan-Meier (KM) survival plots were generated to compare OS among these subtypes. The log-rank test was used, and p-values were corrected using the FDR correction method. Finally, univariate and multivariate Cox regression analyses, integrating subtypes with clinical information, were performed to evaluate the independent impact of subtypes on patient OS.

To further analyse microenvironmental differences among subtypes, we obtained 200 hypoxia-related genes from the HALLMARK_HYPOXIA gene set provided by the Molecular Signature Database (MSigDB). We quantified the extent of hypoxia in each sample using single-sample gene set enrichment analysis (ssGSEA) to calculate a hypoxia score (21, 22). We also analysed the distribution of SUVmax values from ^18^F-FDG PET/CT images. We then estimated the immune cell composition of each sample using the CIBERSORTx (23) deconvolution algorithm on the online platform (https://cibersortx.stanford.edu/). The Impute Cell Fragments job was run in relative mode using the recommended standard LM22 features. Quantile normalization was disabled, and 500 permutations were performed.

### 2.3 Imaging preprocessing and radiomics feature extraction

We applied the preprocessing workflow to data obtained from both the TMUCIH and the Radiomics cohorts using 3D Slicer software (version 5.6.0).

The Radiomics cohort comprised a total of 115 PET/CT data downloaded from TCIA. According to the project description, these imaging data were collected over decades from multiple institutions using various scanner from GE, Philips, and SIEMENS. This study prioritized the use of low-dose unenhanced CT scans performed simultaneously with PET. However, some cases in the TCGA-LUAD project only included iodine-enhanced CT images. Considering that previous studies have reported the stability of most radiomic features between enhanced and unenhanced images, we applied the same normalization and resampling to both image types to avoid introducing additional bias.

For the TMUCIH cohort, the scanning process was as follows: patients fasted for approximately 6 hours before imaging, and their blood glucose levels were controlled to<7.0 mmol/L. ^18^F-FDG was produced using a GEMINI trace medical cyclotron, with a radiochemical purity of >95%. The injection dose was 4.2 MBq/kg, calculated based on patient weight. Image acquisition began 50–60 minutes after injection. A GE Discovery Elite or Discovery 690 PET/CT scanner was used. Low-dose CT (current 50–80 mA, voltage 120 kV) was used for attenuation correction. PET scans were performed from the distal femur to the top of the skull, with a scan time of 2 minutes per scan position, for a total of eight positions. Image reconstruction was performed using an iterative method.

Based on previous studies and the IBSI preprocessing strategy (24–26), we used a cubic spline interpolation algorithm to resample images. The CT images were resampled to a voxel size of 1 × 1 × 1 mm and underwent grayscale normalization. As for PET images, the images were resampled to a voxel size of 3 × 3 × 3 mm and converted to SUV values using Equation 1:

(1) $$SUV=\frac{Pixel\;value*Scale\;Factor*Decay\;Correction}{Radionuclide\;Total\;Dose\left(MBq/kg\right)/Weight\left(kg\right)}$$

The data required for these calculations were extracted from DICOM images. Additionally, images from Philips scanners were converted to SUV values using the formula provided in the instructions.

Some cases in TCGA-LUAD had lesion segmentation provided by TCIA, for the remainder, semi-automatic segmentation was generated using the 3D Slicer software (24). All these segmentations were manually corrected by a radiologist with five years of experience to ensure that the region of interest(ROI) included only tumor lesions while excluding normal lung tissue, chest wall, and mediastinal structures. To assess the reproducibility and objectivity of ROI segmentation, 30 samples were independently corrected by another radiologist with over ten years of experience. The consistency of the ROI delineations between the two radiologists was assessed using the DICE coefficient. The intergroup reliability of the radiomic features extracted from the two ROIs was assessed using a two-way randomized intraclass correlation coefficient (ICC) analysis.

Radiomics features were derived from definitions provided by PyRadiomics and extracted using the Radiomic v3.1.0 in Python 3.9.10. Voxel intensity values were discretized by using a bin width = 10 HU for CT and 0.25 SUV for PET images. The basic feature set includes 14 geometric shape features, 18 first-order pixel value distribution statistical features (FirstOrder), and 68 high-order texture features based on grayscale statistics (22 Gray Level Co-occurrence Matrix (GLCM) features, 16 Gray Level Run Length Matrix (GLRLM) features, 16 Gray Level Size Zone Matrix (GLSZM) features, and 14 Gray Level Dependence Matrix (GLDM) features). Features were extracted from the original image and the following derived images: Laplacian of Gaussian (LoG) filtered images (due to different resolutions, sigma values of 1.0, 2.0, 3.0, 4.0, and 5.0 were used for CT images, and 3.0, 4.0, and 5.0 were used for PET images), wavelet transformed images, gradient images, square images, square root images, logarithmic images, and exponential images. Geometric features were extracted only from the original image. All the features were normalized by using the Z-score algorithm for analysis.

### 2.4 Radiomics-based classifier development

To assess potential batch effects, we labelled batches according to project and scanner manufacturer within the Radiomics cohort, and between the TMUCIH and the Radiomics cohort. PCA was used to visualize dimensionality reduction. We further calculated the silhouette score using the Euclidean distances of the first five principal components to quantify the degree of batch separation. Permutational multivariate analysis of variance (PERMANOVA)was used to test for the significance of batch-related variance.

The Radiomics cohort (n=115) was randomly partitioned into training (70%) and testing (30%) sets. A multi-class classification model was developed using the TabNet classifier (25) (implemented via pytorch-tabnet in Python 3.9.10). Training proceeded for a maximum of 200 epochs, incorporating an early stopping mechanism to prevent overfitting. Hyperparameter optimization was performed using 3-fold nested cross-validation on the training set. During the pre-training and training process, to reduce the dimension of features and compensate for class imbalance, Sparse Canonical Correlation Analysis (sCCA) was performed to select radiomic features significantly correlated with immune-related gene expressions (26). These selected features were fed into a XGBoost model and a Lasso model to identify those with significant importance. The filtered data were subsequently augmented using the Synthetic Minority Over-sampling Technique (SMOTE) and Gaussian Noise Injection.

Model performance was evaluated on the testing set using one-vs-rest AUC, Receiver Operating Characteristic (ROC) curves and standard classification metrics, the 95% Confidence Intervals (CIs) estimated using 1,000 bootstrap iterations. To enhance model interpretability, we analyzed the attention scores assigned to each feature during the sequential decision steps, and assessed the differential contribution of features across subtypes.

### 2.5 Evaluation of predicted subtypes in the independent TMUCIH cohort

We applied the model on to TMUCIH cohort to predict the subtype labels, and based on the result, we obtained 60 tissue samples from the TMUCIH cohort to conducted IHC staining for key immune markers (20 per predicted subtype). Tissue sections, 2-3 μm thick, were obtained from paraffin-embedded tumor sections and stained with primary antibodies against PD-L1 (Proteintech), CD3 (Thermo Fisher), CD8 (Jinqiao Bio), Foxp3 (Abcam), CD11c (Abcam), CD68 (Thermo Fisher), and CD163 (Thermo Fisher). Tissue sections were digitized using a DP controller (Olympus Corporation). Two independent pathologists, blinded to the clinical data of the patients, selected five non-overlapping, non-contiguous fields per slide. The number of PD-L1+ tumor cells, CD3+ TILs, CD8+ TILs, Foxp3+ Tregs, CD11c+ DCs, CD68+ TAMs, and CD163+ TAMs was quantified at an area of ×400 (0.0484 mm²). The mean of the counts was obtained from all five different regions. For subsequent statistical analysis.

In both the TKI-treated and ICI-treated cohorts, multivariate Cox regression analyses were performed to validate the independent impact of subtypes on patient OS (assessed at 1825 days (5 years)). KM survival plots were generated to compare OS among the different subtypes.

### 2.6 Statistical information

Statistical analysis and visualization were performed using R version 4.5.0. Normality was assessed using the Kolmogorov-Smirnov test. For non-normal data, the Kruskal-Wallis test followed by Dunn’s test was applied for multi groups. Kaplan-Meier curves were compared using the Log-rank test with Benjamini-Hochberg FDR correction. Missing values were handled via mean imputation. Multicollinearity in Cox models was assessed using the variance inflation factor (VIF), with a threshold of<5. The Proportional Hazards (PH) assumption was verified using Schoenfeld residuals. The statistical significance criterion for all comparisons was p-value< 0.05.

## 3 Results

### 3.1 Transcriptomic clustering identifies three prognostically distinct LUAD subtypes

**Figure 2:**
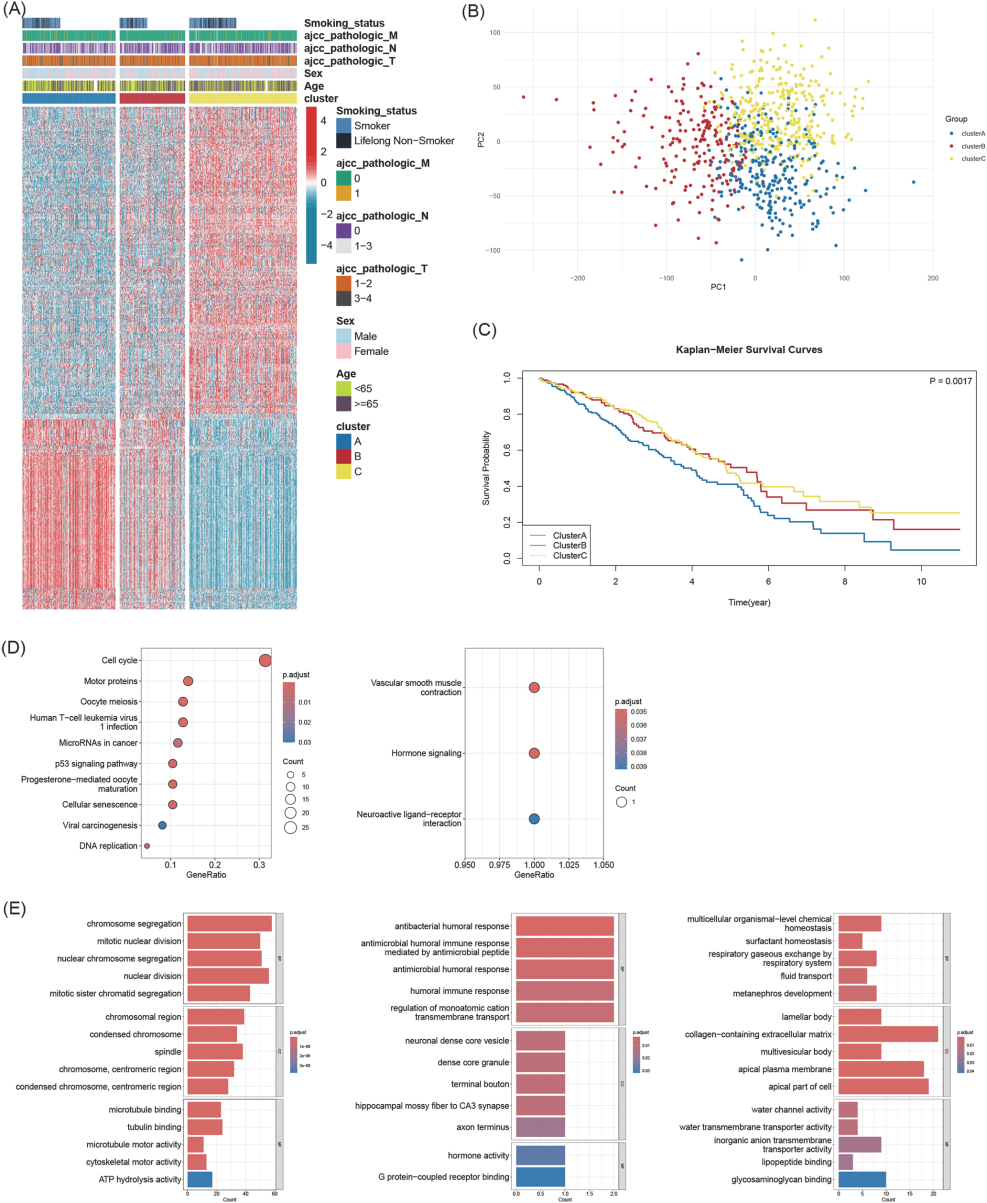
Identification and characterization of three subtypes. **(A)** Heatmap of 407 differentially expressed genes across subtypes. **(B)** PCA plot illustrating the transcriptomic separation of the three clusters. **(C)** KM curves for overall survival (log-rank p-value = 0.0017). **(D)** Significantly enriched KEGG pathways identified in ClusterA and ClusterB. ClusterC showed no significant enrichment. **(E)** Top 5 enriched GO terms (BP, CC, and MF) for each subtype.

Unsupervised consensus clustering of 773 LUAD transcriptomes robustly identified three distinct subtypes, hereafter designated ClusterA, ClusterB, and ClusterC (**Figure 1B**). PCA confirmed clear separation among the subtypes (**Figure 2B**). DGE analysis revealed unique molecular signatures for each cluster (**Figure 2A**). GO/KEGG analysis showed that ClusterA was enriched for pathways related to cell proliferation; ClusterB was associated with humoral immune responses and ion transport; and ClusterC was characterized by pathways involved in substance transport and tissue fibrosis (**Figures 2D, E**).

The subtypes exhibited distinct clinical characteristics (**Table 1**) and were significantly associated with OS (log-rank p-value=0.0017; **Figure 2C**). ClusterA had a worse survival prognosis than ClusterC and ClusterB. (FDR correction adjusted p-values = 0.0130, 0.0033, 0.7696) Univariate COX regression analysis demonstrated that subtypes and AJCC pathologic T, N, M stage were statistically significantly associated with OS. Multivariate COX regression analysis showed that ClusterC and AJCC pathologic T, N, M stage were independent prognostic factors (with VIF were less than 5; **Table 2**), with a C-index of 0.62 for the full model. The PH assumption showed a global chisq of 9.340 and p-value of 0.096, indicating that the assumption is valid.

**Table 1:** Clinical characteristics of subtypes in the genomic set.

Characteristics	Cluster A (n=270)	Cluster B (n=190)	Cluster C (n=313)	p-value
Age (year)-median (range)	63 (38-88)	64.3 (35-87)	67 (36-87)	4.54×10^-05^*
Sex (male%)	62.80%	58.82%	44.73%	0.0023*
EGFR				0.3519
- Mutated	11.20%	7.73%	11.51%	
- Wildtype	8.00%	9.66%	8.22%	
- Unknown	80.80%	82.60%	80.26%	
Smoking status (Former/Current Smoker %)	71.27%	70.13%	63.28%	0.3892
AJCC_pathologic_T (T1-2%)	89.54%	86.63%	88.81%	0.4312
AJCC_pathologic_N (N0%)	60.48%	63.28%	71.04%	0.0132*
AJCC_pathologic_M (M0%)	94.00%	98.06%	95.63%	0.0263*

*For statistically significant (p-value <0.05).

**Table 2:** Univariate and multivariate cox regression analysis of clinical characteristics and subtypes for overall survival.

Characteristics		Genomic set (n=773)					TMUCIH cohort			
							TKI-treated cohort (n=118)		ICI-treated cohort (n=75)	
		Univariate analysis		Multivariate analysis			Multivariate analysis		Multivariate analysis	
		HR (95% CI)	p-value	HR (95% CI)	p-value	VIF	HR (95% CI)	p-value	HR (95% CI)	p-value
Cluster	Cluster A	Ref								
	Cluster B	0.6632 (0.4877-0.9017)	0.0088*	0.8022 (0.5845-1.1009)	0.1724	1.3148	1.2674 (0.6397-2.5113)	0.4969	0.2632 (0.0930-0.7446)	0.0119*
	Cluster C	0.6335 (0.4821-0.8324)	0.0010*	0.7293 (0.5516-0.9642)	0.0267*	1.3150	1.2883 (0.6817-2.4348)	0.4354	0.5310 (0.2009-1.4033)	0.2017
Age (year)	<65	Ref								
	>=65	1.0997 (0.8645-1.3990)	0.4389							
Sex	Female	Ref								
	Male	1.0774 (0.8477-1.3693)	0.5424							
EGFR	Mutant	Ref								
	Wildtype	1.1089 (0.7166-1.7162)	0.6426							
Smoking status	Never Smoking	Ref								
	Former/Current Smoker	1.4461 (0.9426-2.2186)	0.0912							
AJCC_pathologic_T	1-2	Ref								
	3-4	1.766 (1.285-2.4268)	0.0005*	1.5505 (1.1247-2.1375)	0.0074*	1.0113	1.5657 (0.8947-2.7398)	0.1164	0.8544 (0.4271-1.7091)	0.6563
AJCC_pathologic_N	0	Ref								
	1-3	2.0575 (1.6126-2.6251)	6.48×10^-09^*	1.8795 (1.4631-2.4145)	7.91×10^-07^*	1.0213	2.1754 (1.0699-4.4233)	0.0318*	2.2941 (0.8522-6.1752)	0.1003
AJCC_pathologic_M	0	Ref								
	1	1.9415 (1.1687-3.2255)	0.0104*	1.7637 (1.0404-2.9897)	0.0351*	1.0053	1.8319 (0.9332-3.5960)	0.0786	5.7956 (2.1916-15.3262)	0.0004*

VIF, variance inflation factor (VIF > 5 indicates collinearity); HR, Hazard Ratio; CI, Confidence Interval.

### 3.2 Analysis of the overall tumor microenvironment

Immune cell deconvolution using CIBERSORTx revealed significant differences in the composition of 20 out of 22 immune cell types across the subtypes, as well as hypoxia score and SUVmax value (Kruskal-Wallis test and Dunn’s test p-value< 0.05, except for Tregs and eosinophils), confirming they represent distinct immune states.

**Figure 3:**
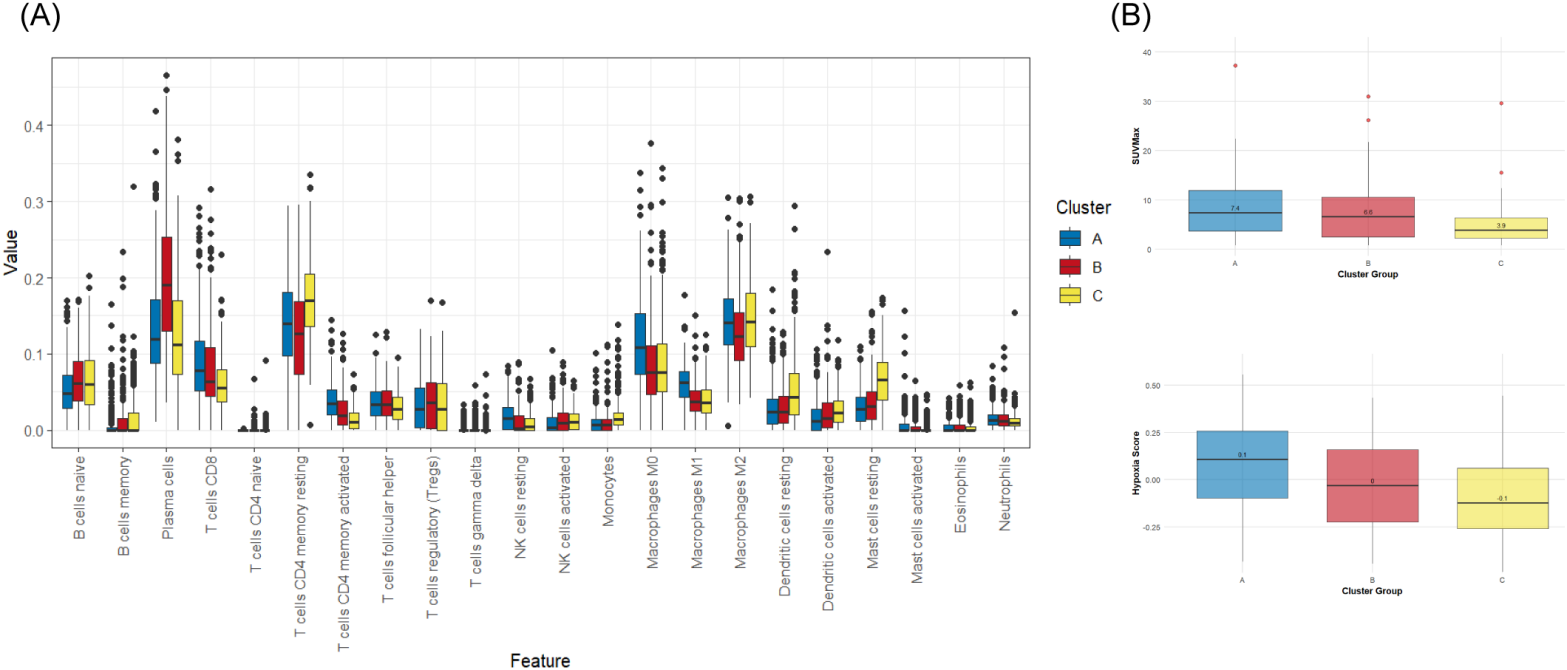
Immune microenvironment analysis across subtypes. **(A)** Box plots showing the estimated immune cell abundance by CIBERSORTx in the Genomic cohort. **(B)** Box plots illustrating the differences in hypoxia scores and SUVmax values (from PET/CT) across three subtypes.

ClusterA showed the highest hypoxia score, highest SUVmax value and elevated expression of CD274, suggesting an exhausted immune phenotype and we termed it Inflammatory. ClusterB termed Activated because of increased humoral immune cells and lowest expression of CD274. ClusterC termed Evasive for highest resting cells and lowest hypoxia levels (**Figure 3**).

### 3.3 Development and performance of radiomics-based classifier

The average DICE coefficient of ROI segmentation results of the two radiologists was 0.863, and the average ICC for a total of 3124 radiomic features (1476 from PET images and 1648 from CT images) was 0.94 (95%CI 0.83-0.97), indicating good inter-observer agreement across both imaging modalities, and only features with an ICC > 0.85 were retained.

**Figure 4:**
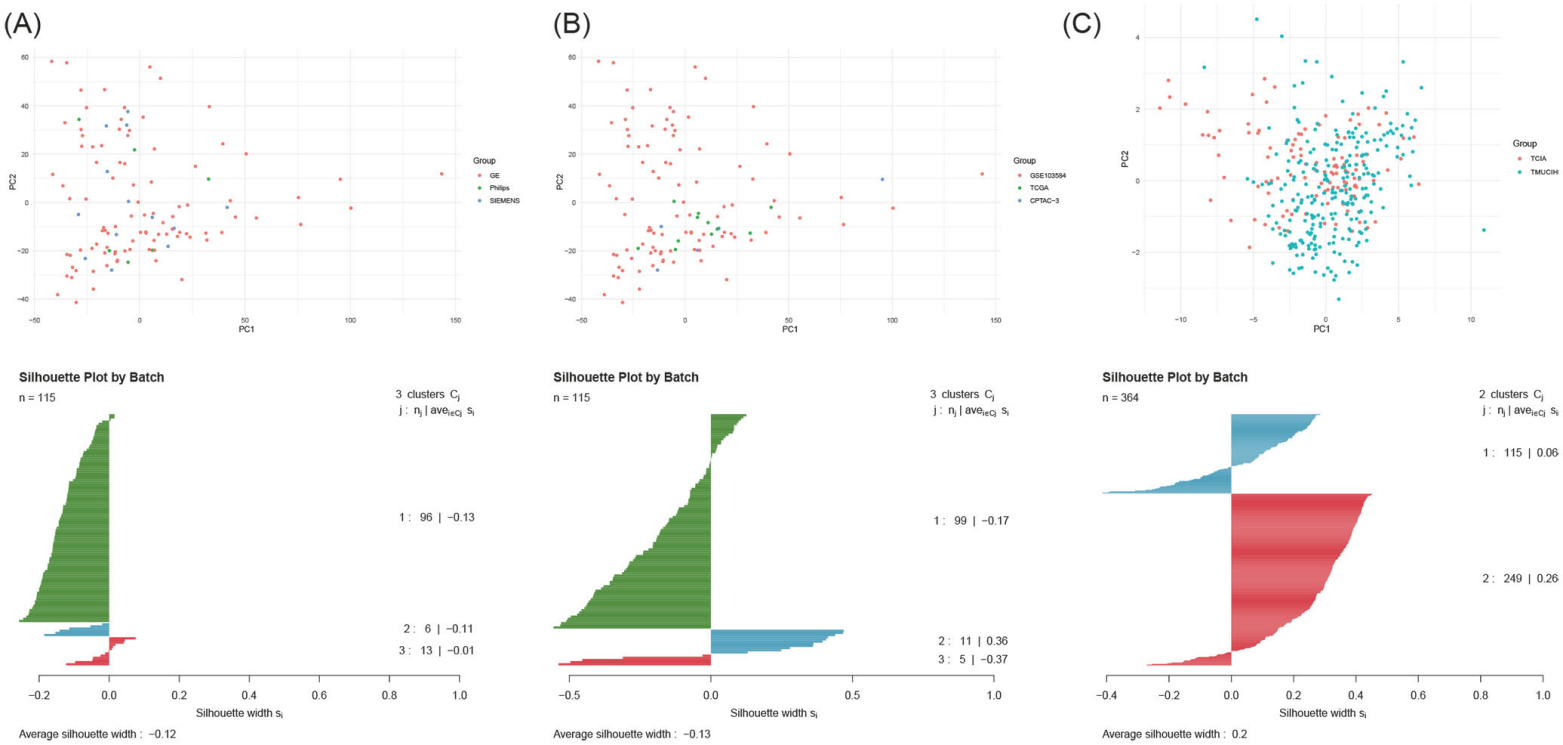
Assessment of batch effects in radiomic features. **(A–C)** PCA plots and corresponding silhouette coefficient analyses.

We found no significant batch effects related to scanner manufacturer or data source within the Radiomics cohort (PERMANOVA p > 0.85, average silhouette score<0, **Figures 4A, B**; **Supplementary Table 2**), supporting the feasibility of building a generalizable model.

**Figure 5:**
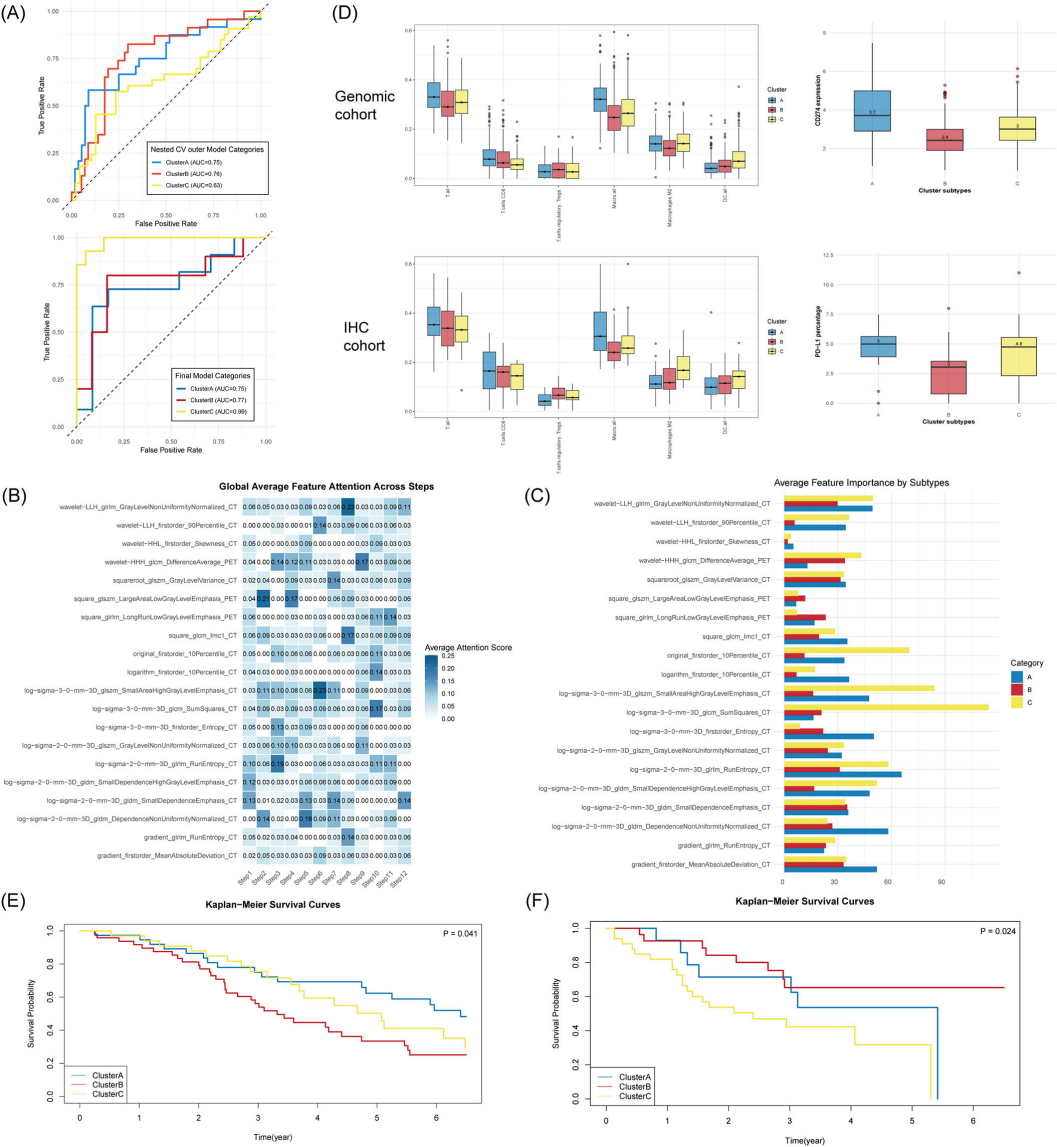
Interpretability and performance of the deep-learning model, and its evaluation on the TMUCIH cohort. **(A)** ROC curves for mean outer fold model of nested cross-validation and the final model. **(B)** Heatmap of the average attention scores to each radiomics features across sequential decision-making steps in the model. **(C)** Bar plots show differential contribution of radiomics features across subtypes. **(D)** Comparison of Z-score normalized immune cell infiltration and PD-L1 levels between the Genomic cohort (upper row) and the IHC cohort (lower row). IHC markers were mapped to CIBERSORTx cell types as follows: CD8+ TILs (T.cells.CD8), Foxp3+ Tregs (T.cells.regulatory.Tregs.), CD3+ TILs (T.all), CD11c+ DCs (DC.all), CD68+ TAMs (Macro.all), and CD163+ TAMs (Macrophages.M2). **(E, F)** KM curves for the TKI-treated cohort and ICI-treated cohort.

The prediction results of the outer fold model of nested cross-validation were merged, and the macro One-vs-Rest AUC was 0.71. classification AUCs were 0.75 (95% CI: 0.63-0.88), 0.76 (95% CI: 0.64-0.88), and 0.63 (95% CI: 0.50-0.76) for ClusterA, ClusterB and ClusterC). The final model, based on the former parameter settings, uses 20 features and 12 decision steps, achieved an average accuracy of 0.80 and a one-vs-rest AUC of 0.84 (**Figure 5A**; **Table 3**) on the test set. The model showed particularly strong performance in identifying ClusterC (classification AUCs were 0.75(95% CI: 0.55-0.95), 0.77(95% CI: 0.57-0.98), and 0.99(95% CI: 0.96-1.00) for ClusterA, ClusterB and ClusterC). The attention scores assigned to each feature during the sequential decision steps showed in **Figure 5B**, and the contribution of features across subtypes was showed in **Figure 5C**.

**Table 3:** Performance evaluation of radiomics models.

Real subtypes	Predicted cluster A	Predicted cluster B	Predicted cluster C	Precision	Recall	F1-score
Actual Cluster A	7	3	1	0.7778	0.6364	0.7000
Actual Cluster B	2	8	0	0.6667	0.8000	0.7273
Actual Cluster C	0	1	13	0.9286	0.9286	0.9286
accuracy	0.8000 (95% CI: 0.6571 - 0.9143)					
macro avg				0.7910 (95% CI: 0.6429 - 0.9231)	0.7883 (95% CI: 0.6402 - 0.9185)	0.7853 (95% CI: 0.6297 - 0.9126)

### 3.4 Evaluation on the TMUCIH cohort

Statistical analysis revealed no significant batch effects in radiomic features between the Radiomics and TMUCIH cohorts (PERMANOVA p=0.155, average silhouette score was 0.200, **Figure 4C**; **Supplementary Table 2**), confirming the stability of the features across different centers.

The immune cell densities and PD-L1 level in the predicted subtypes closely mirrored the patterns observed in the transcriptomic-defined clusters, providing strong evidence that our non-invasive model captures true biological heterogeneity (**Figure 5D**). Then we assessed clinical validity in two distinct treatment settings. In the TKI-treated cohort (n=118), the subtypes showed a clear trend towards prognostic stratification (log-rank p-value =0.041; **Figure 5E**). Interestingly, ClusterA exhibited a favorable survival trend here. However, this difference did not retain statistical significance after multiple-testing correction (FDR-adjusted p-value=0.058, 0.279, 0.252). In the multivariable analysis for this cohort, only pathologic N-stage was an independent prognostic factor. In the ICI-treated cohort (n=75), KM curves showed statistically significant differences in OS among the three subtypes (log-rank p-value = 0.024). Multivariate Cox regression identified ClusterB status and AJCC pathologic M-stage as independent prognostic factors for OS. The C-index is 0.69 (**Table 2**; **Figure 5F**).

## 4 Discussion

LUAD remains a critical area of research due to its significant heterogeneity and rapid progression, which contribute to the unsatisfactory overall performance of current therapies. Many studies in LUAD have leveraged public resources to identify immune-related or functionally distinct prototypes, discovering numerous genes or scores associated with clinical prognosis. Radiomic studies often limit themselves to identifying associations between genetic alterations and radiomic features, or integrated only as a component within multi-omics clustering (5, 27–30), thus failing to fully utilize its unique potential. In this study, we employed unsupervised clustering of transcriptome data to delineate three subtypes with unique clinical and immune microenvironmental characteristics. Using radiomic features extracted from ^18^F-FDG PET/CT images, we trained a non-invasive prediction model. The predicted subtypes demonstrated similarity with the transcriptomic subtypes in immunohistochemical patterns, and showed prognostic significance in different clinical treated cohorts.

Our analysis revealed three distinct subtypes each with unique TME compositions and molecular pathway enrichments. ClusterA, despite high levels of cytotoxic T-cells, concurrently showed markers of immune exhaustion (CD274 upregulation) and hypoxia, portraying a state of dysfunctional inflammation. ClusterB was primarily enriched in humoral immune response pathways, low myeloid cells, and the lowest CD274 expression, collectively suggesting a highly activated immune state. In contrast, ClusterC displayed features of a metabolically quiescent and fibrotic TME. These distinct subtype characteristics do not fully align with traditional cold/hot tumour classifications and reveal complex, heterogeneous patterns within the immune microenvironment (4, 31).

We observed the prognostic impact of the subtypes was treatment-dependent. In the TKI treated cohort, we observed a trend where the typically poor-prognosis ClusterA showed better outcomes. Although this trend did not reach statistical significance after correction, possibly due to sample size, this reversal is generating a hypothesis and consistent with the notion that EGFR-mutant NSCLCs typically present with low tumour mutational burden and a less inflammatory tumour microenvironment (32, 33). Subsequently, in the ICI-treated cohort, we observed that ClusterB responded favorably to ICI therapy, consistent with its abundant immune reserves. The lowest CD274 expression/PD-L1 level may indicate low and easily reversible immunosuppression. Conversely, ClusterA displayed more severe immunosuppression, but it still showed a better prognosis than ClusterC, possibly because ClusterC experienced upregulation of fibrosis and angiogenesis-related pathways, which likely impairs antigen presentation and anti-tumor immune responses. This differential response underscores that the efficacy of immunomodulatory strategies hinges on a baseline immune response and the release of pre-existing immunity, mediated by multifaceted mechanisms within the tumour immune microenvironment (34–37).

Limitations of this study have been the retrospective nature and inherent biases. We were unable to control the image reconstruction algorithm. While batch effect mitigation, voxel resampling and normalization was employed to harmonize the data, cross-platform inconsistencies in imaging acquisition may still affect feature reproducibility. Despite using data augmentation and nested cross-validation for parameter tuning, the small sample size may have led to unstable results. Our final model excelled at identifying ClusterC, reflecting a distinct radiological phenotype. However, it faced challenges in differentiating ClusterA from ClusterB. This ambiguity likely stems from either the shared immunological continuity between these subtypes or the class imbalance within our testing set. Future studies utilizing larger, more balanced datasets may help resolve these disparities. Our genomic cohort was primarily of Western ancestry. Given the known ethnic disparities in genomic landscapes, such as EGFR mutations between Caucasian and East Asian NSCLC populations (38–40). Our evaluation in TKI-treated cohort may exist some inherent biases, which require validation in more diverse ethnic groups. And the sample sizes in our sub-cohorts were limited. While our results are promising, particularly in the ICI cohort. Definitive assessment of its prognostic value will require validation in larger, multi-center cohorts.

## 5 Conclusion

We identified three immune subtypes in LUAD and developed a non-invasive ^18^F-FDG PET/CT radiomics tool for their prediction, which conducted a biologically and clinically supported external evaluation. Our work demonstrated a framework for linking genomics to radiology in LUAD. This provides some new possibilities for achieving accurate stratification of the disease, understanding its biological heterogeneity, and ultimately guiding the development of personalized treatment strategies.

## Data Availability

The raw data supporting the conclusions of this article will be made available by the authors, without undue reservation.

## Supplementary material

Supplementary Table 1 Provides a comprehensive overview of all the cohort data.

Supplementary Table 2 Provides the PERMANOVA test results among different batch in TMUCIH and the radiomics cohort.
